# Visual processing of color and shape in people with leprosy: Visual processing in people with leprosy

**DOI:** 10.1186/s41155-020-00153-w

**Published:** 2020-07-18

**Authors:** Perla Figueredo Carreiro Soares, Michael Jackson Oliveira Andrade, Sefora Luana Evangelista Andrade, Natanael Antonio Santos

**Affiliations:** 1 Neurosciences and Behavior, Department of Psychology, Universidade Federal da Paraíba, João Pessoa, Paraíba, Brasil; 2 Psychology, Department of Psychology, Universidade Federal da Paraíba, João Pessoa, Paraíba, Brasil; 3 Nursing, Department of Nursing, Universidade Federal da Paraíba, João Pessoa, Paraíba, Brasil

**Keywords:** Contrast sensitivity, Color confusion axles, Leprosy

## Abstract

The aim of this study was to analyze the contrast sensitivity of spatial luminance and the color discrimination thresholds of the protan, deutan, and tritan axes of people with leprosy. This study included 8 subjects with leprosy (M = 4, W = 4, M = 33.38 ± 8.7) and 8 healthy subjects (M = 4, W = 4, M = 30.89 ± 5.8). The contrast sensitivity was evaluated by the Metropsis software version 11.0 with vertical sinusoidal grids of frequencies of 0.2, 0.5, 1, 5, 10, and 16 cycles per degree of visual angle (cpd) and color vision by the desaturated Lanthony D15 tests and the trivector and ellipse protocols of the Cambridge Color Test. The results showed significant differences between the groups in the processing of spatial frequencies of 0.2 (*U* = 14; *p* = .018); 5.0 (*U* = 45.0; *p* = .001); 10.0 (*U* = 45.0; *p* = .001), and 16.0 (*U* = 45.0; *p* = .001) cpd. The difference in color recognition through D15d (*U* = 4.0; *p* = .002). Ellipse 2 (*U* = 10.0; *p* = .012) and ellipse 3 (*U* = 9.0; *p* = .009) were discriminated against. Overall, the results indicate that leprosy changes the visual processing of low, medium, and high spatial frequencies, as well as the sensitivity of the short wavelength (tritan line of confusion) and long (protan line of confusion) cones.

## Introduction

Leprosy is an infectious disease caused by *Mycobacterium leprae* and can be considered a neurological disease (Brasil, 2018). Leprosy is classified into two forms, paucibacillary and multibacillary, and the paucibacillary form is subclassified in the indeterminate and tuberculoid clinical forms, which are characterized mainly by a low bacillary load (Brasil, 2002). However, multibacillary leprosy can be classified as virchovian and dimorphic, having a high bacillary load capable of presenting positive bacilloscopy and, therefore, it is the transmitting form of the disease. (Brasil, 2002; Simpson et al., 2010; Ribeiro et al., 2018). The affinity of the Hansen’s bacillus with neuronal cells causes involvement of sensory, motor, and/or autonomic fibers, causing a demyelinating neuropathy (Bechler, 2011; Mendonça et al., 2008; Rambukkana, 2000; Vieira, Soares, & Silva, 2008).

Studies indicate that leprosy causes impairment in the sensory and perceptual mechanisms of vision (Dethlefs, 1981; Malik et al., 2011). According to Brasil (2008), the nasolacrimal canal would be the access route of the bacillus into contact with structures of the visual system. Based on this assumption, some studies have investigated the prevalence of ocular lesions in people with leprosy and have identified decreased visual acuity (Malik et al., 2011), glaucoma (Souza et al., 2005), cataract (Shrestha et al., 2018; Whitcher et al., 2001), opacity of the cornea (Courtright and Daniel, 2002, Thompson et al., 2006), and blindness (Eballe et al., 2009; Chukka et al., 2011).

Ulvi et al. (2003) evaluated 74 participants (37 with leprosy and 37 healthy pairs for age) with visual potentials evoked (VEP) using a reverse pattern. The authors identified a slow response pattern. Neto et al. (2006) also observed changes in VEP in 13 patients with leprosy, that is, latency values were significantly higher in leprosy patients. These studies indicate that the visual pathways may be affected by the disease.

Daniel et al. (2005) argue that multibacillary and paucibacillary forms cause changes in color discrimination and visual system sensitivity to detect luminance contrast. Daniel et al. (2005) used Vistech VCTS 6500 test units to evaluate the CS of 127 leprosy patients without clinically visible ocular complications and 123 healthy subjects. The authors reported a loss in CS of patients with leprosy in all spatial frequencies tested (1.5, 3, 6, 12, and 18 cpd). Caires et al. (2014) also evaluated the achromatic and chromatic psychophysical performance of 31 leprosy patients with normal visual acuity compared to a control group composed of 28 subjects. In contrast with Daniel et al. (2005), the authors found a significant difference in CS only at the spatial low frequency of .5 cpd. In relation to the Farnsworth-Munsel 100 (FM100) color test, the authors found a greater number of errors in subjects with leprosy, with no specific preference for color axes.

Overall, leprosy research shows impairments in VEP latency and indicate psychophysical changes in visual contrast processing. However, only one study measured psychophysics with Vistech VCTS 6500 cards (Daniel et al., 2005). Thus, psychophysical studies with discrimination of form and color are still scarce. Thus, the present study sought to measure the spatial luminance contrast and color discrimination thresholds of the protan, deutan, and tritan axes of leprosy patients using Metropsis software from Cambridge Research Systems, Rochester, UK.

## Materials and methods

### Locus of the study

The research was carried out in the Laboratory of Perception, Neuroscience, and Behavior. The experimental laboratory is located in the city of João Pessoa (Paraíba, Brazil).

### Participants

Initially, 57 subjects between the ages of 18 and 45 participated in this study, 37 subjects with leprosy and 20 healthy subjects. However, according to the exclusion criteria, only 8 subjects with leprosy (M = 4, W = 4, M = 33.38, SD = 8.7) and 8 healthy subjects (M = 4, W = 4, M = 30.89, SD = 5.8) concluded the study. The diagnosis and notification of leprosy were reported according to the National System of Aggravations and Notifications (SINAN) by professionals of a Reference Hospital in the treatment of Infectious Diseases. Participants with leprosy who had reactions to medication were excluded in order to homogenize the sample. Regarding the healthy subjects, they were considered participants with age, schooling, and sex similar to the group of participants with leprosy.

### Instruments

#### Sociodemographic questionnaire

A questionnaire was used with social questions (such as sex, age, time preference, monthly income, among others) and clinical ones (physical activities, psychological and neurological impairments, psychoactive substances consumption, among others) according to the needs of the study.

#### Achromatic vision tests

The achromatic vision evaluation was performed using Metropsis software (Cambridge Research Systems, Rochester, UK) with the contrast sensitivity function (CSF). We used a 19-inch LG monitor, 1280 × 1024 pixels, a VSG 2/5 (Cambridge Research Systems) and a Dell Precision T3500 computer with the W3530 graphics card. The mean luminance used was 34.4 cd/m^2^ and the vertical sinusoidal grid stimuli were presented at 150 cm at spatial frequencies of 0.2, 0.5, 1, 5, 10, and 16 cpd. The stimuli had quadric formats with dimensions 5° of visual angle, presented with 2.5° of the center of the screen towards the periphery.

### Measures of color perception

#### Lanthony D-15 desaturated test (D15d)

This test evaluates the ability of the visual system to recognize color through pseudo-isochromatic parts (Good et al., 2005). It is an array arrangement test composed of 15 plastic discs covered by Munsell paper (Lanthony & Dubois-Poulsen, 1973). The test has a reference plate that is fixed and 15 other plates with different shades of the same brightness (8) and saturation (2), with 1.5° of visual angle at a distance of 50 cm (Lanthony 1978). The matrices of sheets form a circle in the Munsell color space; their correct arrangement is indicated by a number on the back of each disk. Only the color confusion index (CCI) value was used as an indicator of how much the total color distance score (TDCS) deviated from the reference value (Geller, 2001).

#### Cambridge color test (CCT)

In this study, we applied the CCT 2.0 version (Cambridge Research Systems, Rochester, UK) and a visual stimulus generator (VSG 2/5; Cambridge Research Systems, Rochester, UK) LG with gamma correction and made with a Color Call photometer (Cambridge Research Systems, Rochester, UK). The test was performed in a dark room binocularly at a distance of 300 cm from the monitor. The Forced Choice method was used with four alternatives (Andrade et al., 2018), starting the excursion at a specific point in the 1976 International Commission on Illumination diagram (Geller & Hudnell, 1997) and extending to the center of the color, *U'V'* space (.197, .469, Costa et al., 2012). Each participant responded with the answer box, the orientation of the chromatic stimulus in the form of C, moving it up, down, to the right, or to the left. For each correct response, the background chromaticity approached the chromaticity of target C (ranging from 5.7 to 2.8 mm in diameter) and for each incorrect response, the chromaticity of both was spaced apart. The absence of a response was considered incorrect and the test was completed when 11 reversals were performed for each axis tested and the contrast threshold was defined after the last six reversals (Lima et al., 2011).

### Procedure

This study was divided into two moments. At the first moment, the clarifications were made on the ethical aspects of the research and the completion of the sociodemographic questionnaire. Then the psychophysical tests were applied. In the achromatic vision test, the dynamic ladder method was used on a logarithmic scale with a three-level variation for the presentation of the stimuli. The participant was at a distance of 150 cm from the screen of the monitor and had the task of responding if the stimulus appeared on the left or right side of the monitor (method of forced-choice with two spatial alterations, 2AFC). Initially, contrast values appeared at the supraluminary level (where the participant was expected to perform a series of correct responses) with the regression criteria of the three-hit scale and an error, for contrast to decrease or increase. Thus, in the series of three consecutive successes, the contrast decreased by 0.7 decibels (dB), and at each wrong response, contrast values increased by 1 dB. After recording 8 reversals (maximum and minimum limit values) for each spatial frequency, the experiment was terminated (Andrade, Santos, Cristino, Oliveira, & Santos, 2018a). Each reversal was defined as a percent value of the stimulus contrast for this trial, and for each spatial frequency, an average of the eight reversals was used as the mean for each participant’s contrast sensitivity. The stimuli had an exposure time of 200 ms, with an interval of 100 ms between them.

The application of the D15d test was performed in a room with 60 w incandescent lighting with 45° angulation and 21 cm distance of a table covered with black cloth. In addition, C-daylight glasses were used to correct incandescent light in a normal proportion relative to daylight. This magnitude allows for more accurate color evaluation results with the D15d test.

In the CCT, the forced-choice psychophysical procedure was used with four spatial alternatives (4AFC). In both protocols, the following procedure was used: the participants had the task of identifying one of the four possible orientations of the opening of the stimulus test “Landolt C” when they appeared on the monitor screen. Each stimulus lasted 300 ms (Mollon & Regan, 2000). The chromaticity of the stimulus varied according to the participant’s response, approaching the background for each correct response. The measurements were also performed in binocular form. In general, each session of the second stage had an average duration of 40 min.

### Ethical aspects

Participation was voluntary and all participants signed the informed consent form. The study was approved by the Ethics Committee (Protocol N. 0660/13, CAAE: 24796113.3.0000.5188). Thus, the present study complied with Resolution (466/12) of the National Health Council, which deals with research guidelines and standards involving human beings and that ensures the confidentiality and anonymity of the data obtained from the participant during and after the research.

### Data analysis

Statistical analysis was performed using the IBM SPSS Version 21.0 software. The descriptive analysis included sociodemographic characteristics and vision measures (mean, median, and standard deviation). Shapiro-Wilk tests showed that visual performance for many tests was not normally distributed. Thus, the differences between groups in the continuous variables were analyzed with the non-parametric Mann-Whitney test. Also, the Spearman’s *σ* test was used to correlate the variables. The 95% confidence interval was used.

## Results

All participants had visual acuity (20/20) and normal color recognition (Ishihara test). The groups did not present a significant difference between age (*p* = .498) and schooling (*p* = .656). Regarding the paucibacillary and multibacillary clinical forms of leprosy, the Mann-Whitney *U* test has not found a significant difference in relation to treatment time (*p* = .051), age of participants (*p* = .297), and schooling (*p* = .207).

Table 1 presents the CSF for healthy subjects and subjects with leprosy. Higher contrast sensitivity values mean greater sensitivity of the participant to the spatial frequency evaluated in the test.

**Table 1:** Distribution of the mean (standard deviation) and median scores of the contrast sensitivity and color discrimination test (Lanthony D15d and CCT)

	Groups						
	Control			Leprosy			*p*
	*M*	SD	Md	*M*	SD	Md	
0.2 cpd	111.78	31.5	111.16	73.85	19.5	84.45	.018*
0.5 cpd	140.82	33.8	157.31	142.10	12.5	141.84	.387
1.0 cpd	182.10	45.5	173.75	168.64	9.9	170.86	.666
5.0 cpd	335.34	63.6	364.54	125.86	55.1	160.32	.001*
10.0 cpd	174.64	34.0	163.53	47.33	21.4	50.5	.001*
16.0 cpd	40.81	12.5	45.66	8.21	3.9	9.03	.001*
CCI	1.07	.1	1.07	1.61	.5	1.38	.002*
Protan	36.53	3.4	37.48	50.30	12.9	47.5	.393
Deutan	44.69	3.3	45.49	60.25	20.1	61.35	.093
Tritan	60.56	9.8	59.00	93.29	17.7	101	.002*
*A1*	409.13	77.8	401.78	703.29	280.8	795.45	.093
*A2*	693.53	202.3	810.5	1188.58	430.1	1173.27	.012*
*A3*	463.52	213.1	517.8	740.35	273.5	732.86	.009*

*Note*: cpd cycle by visual angle degree, *CCI* color confusion index, *A* area of the ellipse, *p < 0.05.

**Fig. 1:**
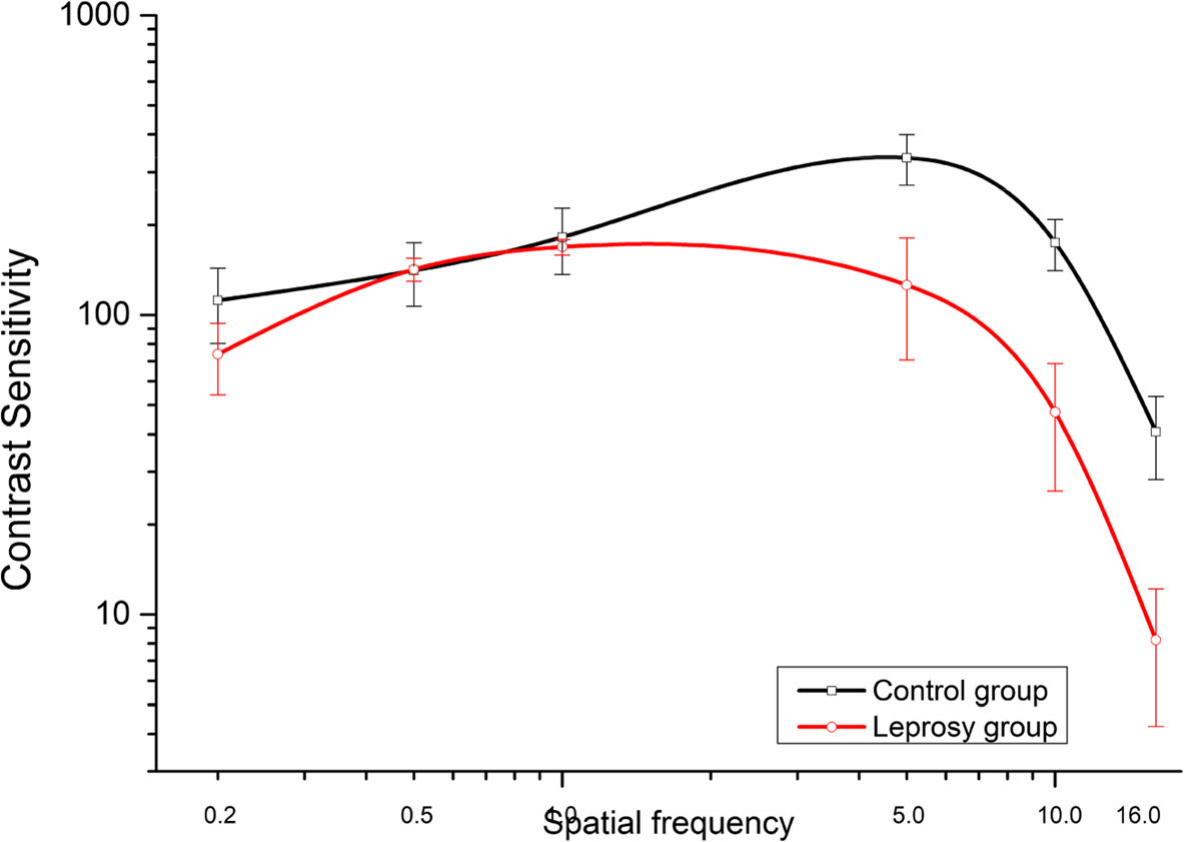
Visual contrast curves plotted as a function of spatial frequencies (cpd) for subjects with normal vision and leprosy

Figure 1 shows the CS data versus the spatial frequency of the two groups. The best fit function shows the following parameters: peak contrast sensitivity at 5 cpd for healthy subjects (M = 335.34) and 1 cpd for subjects with leprosy (M = 168.64), still healthy subjects (M = 182.10) had higher sensitivity in the frequency of 1 cpd than subjects with leprosy. The CSF describes an inverted U-shaped curve with the slope in the low and high frequencies and a sharp slope in the average frequencies. The data analysis with Mann-Whitney *U* test showed a significant difference between the groups for the spatial frequencies of 0.2 (*U* = 14; *p* = .018); 5 (*U* = 45.0, *p* = .001); 10 (*U* = 45.0, *p* = .001); and 16 (*U* = 45.0, *p* = .001) cpd. The control subjects had higher SC in all spatial frequencies than subjects with leprosy (0.2 cpd = 33.94%, 5 cpd = 62.69%, 10 cpd = 72.99%, and 16 cpd = 79.88%). The CS data of leprosy subjects did not present a significant difference in clinical form.

**Fig. 2:**
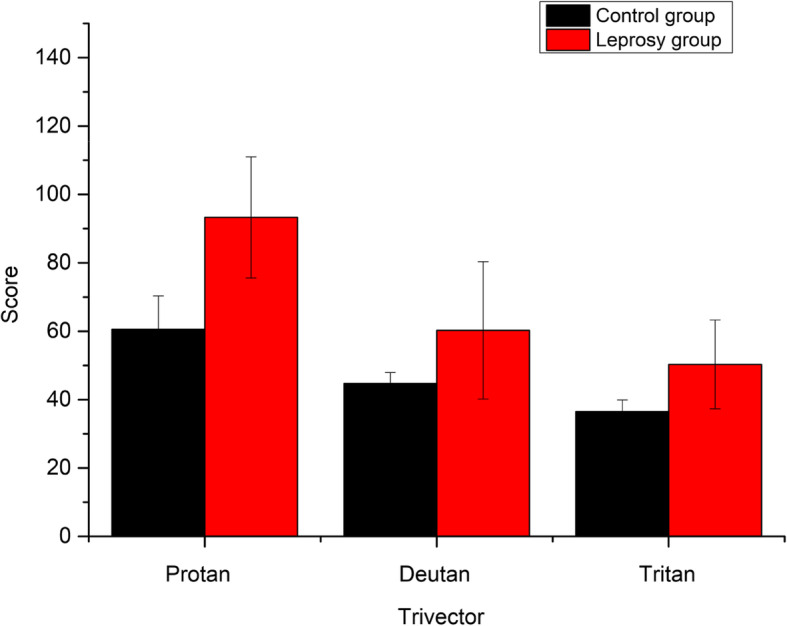
Mean color confusion index (CCI) results for subjects with normal vision and leprosy

In CCI, subjects with leprosy presented higher scores (M = 1.61 ± .5) than controls (M = 1.07 ± .1) the Mann-Whitney test should significant difference in the Lanthony D15d test (*U* = 4.0, *p* = .002, Fig. 2); thus, the proportion of error committed by subjects with leprosy was 33.55%. The CCI scores of subjects with leprosy correlate with the treatment time (*σ* = 0.82, *p* = .012).

**Fig. 3:**
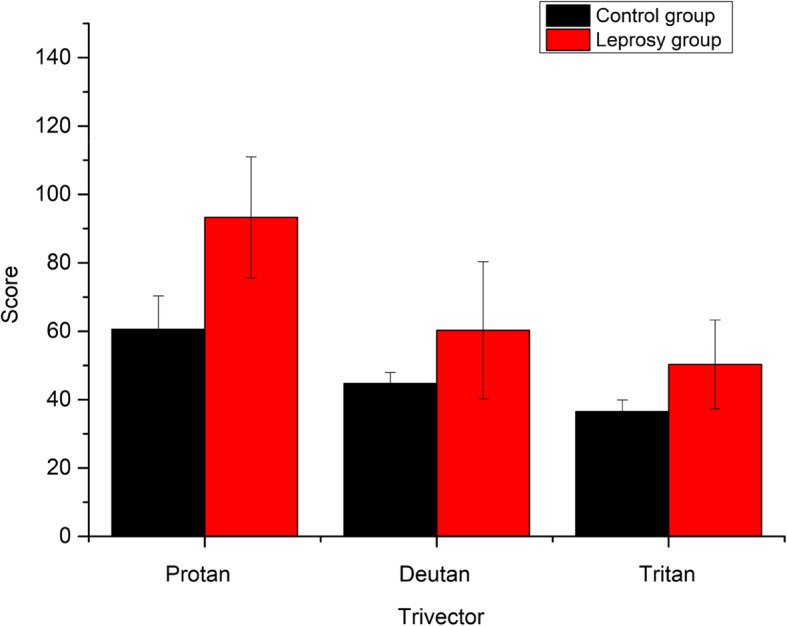
Mean results of the Trivector protocol (*U'V'* × 10^3^) for subjects with normal vision and leprosy

There was also a higher mean score for leprosy subjects in the CCT trivector protocol (Table 1). The Mann-Whitney *U* test indicated significant differences between subjects with leprosy (M = 93.29 ± 17.7 × 10^3^*U'V'*) and control (M = 60.56 ± 9.8 × 10^3^*U'V'*) only for the tritan axis of confusion (*U* = 4.0, *p* = .002, Fig. 3). The tritan axis scores were not correlated with age (*p* = .062) and treatment time (*p* = .466). The data also showed no difference in the axes of confusion and paucibacillary and multibacillary forms.

**Fig. 4:**
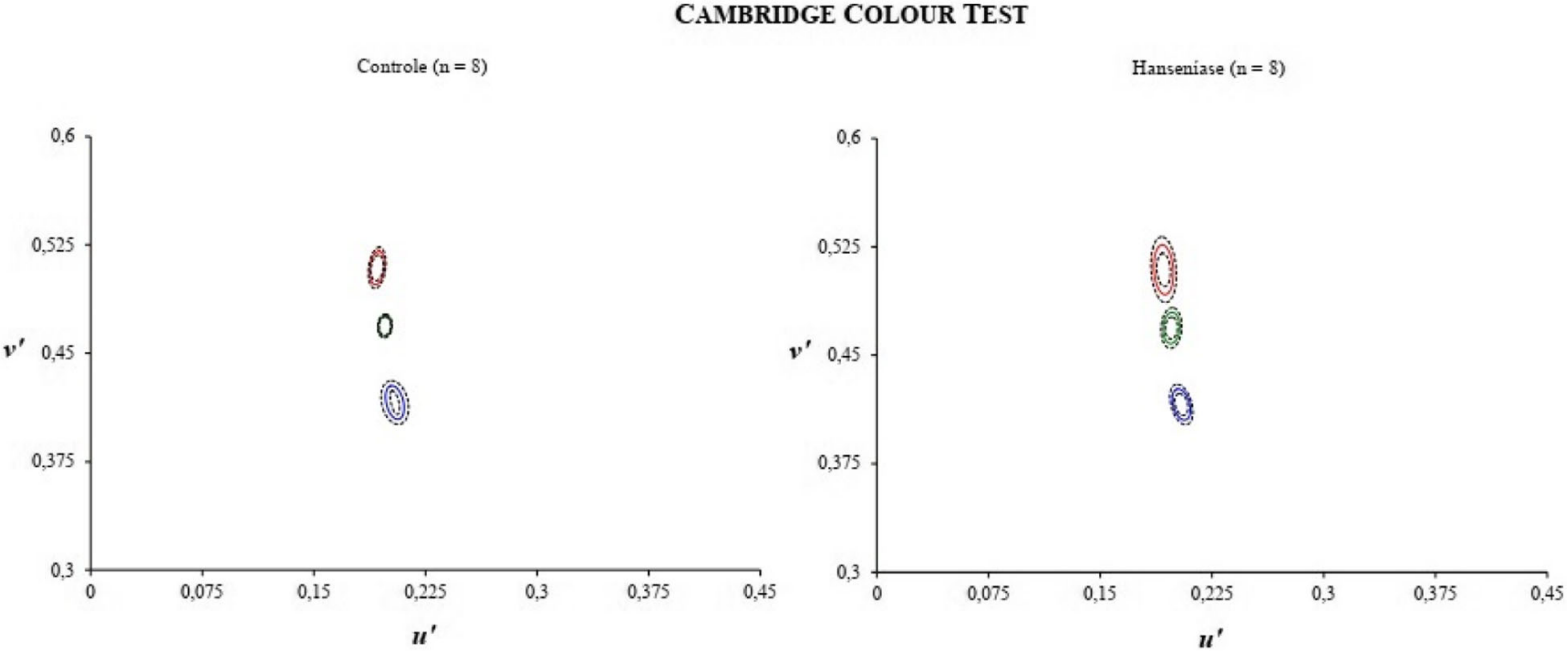
Color discrimination thresholds assessed by CCT expressed as MacAdam ellipse areas in units of (*U'V'* × 10^−6^) for subjects with normal vision and leprosy

The ellipsis protocol from the Mann-Whitney *U* test showed a significant difference for the areas of ellipse 2 (*U* = 10, *p* = .012) and ellipse 3 (*U* = 9.0, *p* = .009). In relation to ellipse 2, subjects with leprosy (M = 1188.58 ± 430.1 × 10^−6^*U'V'*) presented greater area than healthy subjects (M = 693.53 ± 202.3 × 10^−6^*U'V'*). Still, ellipse 3, subjects with leprosy (M = 740.35 ± 273.5 × 10^−6^*U'V'*) also presented a greater area than healthy subjects (M = 463.52 ± 213.1 × 10^−6^*U'V'*). Subjects with leprosy presented an area of 41.65% and 37.39% in elipers 2 and 3, respectively, higher than healthy subjects (Fig. 4). None of the areas correlated with treatment time and age. The data also did not present differences in the areas of the ellipses and the paucibacillary and multibacillary forms.

## Discussion

Leprosy is a chronic disease with disabling complications in the visual system. The aim of our study was to measure the contrast sensitivity of achromatic and chromatic luminance to verify possible changes in visual sensitivity for achromatic sinusoidal grids and color discrimination thresholds of the protan, deutan, and tritan axes of people with leprosy. The contrast sensitivity (CS) evaluates the ability of the visual system to discriminate spatially modulated contrast levels against a homogeneous gray background pattern (Andrade, Neto, Oliveira, Santana, & Santos, 2018b; Santos, Simas. & Nogueira, 2003). The color discrimination measures the ability of the visual system to discern colors from thresholds for short (S), medium (M), and long (L) wavelengths (Andrade et al. 2018b; Lima, Gomes, Ventura, & Silveira, 2011; MacLeod, 2003).

We found SC losses in the low (0.2 cpd) and high (5, 10, and 16 cpd) spatial bands, but we did not find any losses in the low and medium frequency bands (0.5 and 1.0 cpd), according to Fig. 1. These results corroborate with the results of the only study found in the literature by Daniel et al. (2005) who measured SC with Vistech VCTS 6500 cards and reported differences in CS of leprosy patients at spatial frequencies of 1.5, 3, 6, 12, and 18 cpd. Daniel et al. did not measure CS from leprosy patients at spatial frequencies below 1.5 cpd, different from our study that measured CS at lower frequencies. In comparison with Caires et al. (2014), this study also pointed out differences in the sensitivity to low frequency, specifically 0.2 cpd. However, it did not show any significant difference in average frequency (1.0 cpd). Santos and Simas (2003) argue that the human visual system has maximum sensitivity for this type of spatial frequency, so, because it has maximum sensitivity, they may be the last frequency filters to be modified by the active bacillus. Overall, leprosy subjects needed 62% more contrast on average than healthy subjects at all tested spatial frequencies.

We also found for the first time impairments in color recognition using the ICC of leprosy patients with the Lanthony D15d test (Fig. 2). As we found, significant losses in the chromatic luminance sensitivity for patients with leprosy in the trivector protocol of the CCT (Table 1) for the tritan confounding axis (Fig. 3) and for the protocol of ellipses in the ellipse 2 and ellipse 3 areas. This study used the Lanthony D15d test and showed that the leprosy subjects made about 33.55% more errors than healthy subjects. Errors in the ICC correlated with damages to the tritan confounding line (related to short wavelengths). The higher the threshold and area values, the greater the confusion on chromaticity lines. According to Ventura et al. (2009), the major defects acquired in clinical specimens occur on the blue-yellow axis.

Despite the limited number of studies on the impact of abnormal color vision, the results pointed the effects of leprosy on the chromaticity lines of the short (tritan fuzzy line) and long (protan fuzzy line) cones. Just as the active bacillus can measure luminance-sensitive spatial frequency filters, they can also alter photosensitive visual pigments responsible for absorbing wavelengths that enable color vision. In this study, the results have not presented differences in relation to paucibacillary and multibacillary clinical forms, but it is assumed that the sample was small.

It is not possible to make a direct comparison between our results and other results in the literature since the psychophysical studies with CS of achromatic and chromatic luminance are still rare and this is the first time that the basic visual functions for object and color with the method and using Metropsis software (Cambridge Research Systems, Rochester, UK). However, these results were somewhat expected considering the literature that shows neurophysiological, sensorial, and perceptual alterations (Dethlefs, 1981; Malik et al., 2011), alterations in the latency of visual evoked potentials (Ulvi et al., 2003) and psychophysical alterations in CS with the Vistech VCTS 6500 cards (Daniel et al., 2005). Thus, psychophysical studies with discrimination of form and color are still scarce, favoring the realization of new studies to elucidate how the bacilli of leprosy interact as mechanisms or pathways involved in the processing of achromatic and chromatic luminance contrast.

The effects of leprosy on visual mechanisms, its frequency, and severity, depends on several factors, such as the type of leprosy, the stage of the disease, and the time of its treatment. Therefore, future follow-up research with psychophysical methods should investigate the effects of drugs on neural pathways, and consequently on visual functions.

## Conclusion

Overall, subjects with leprosy require an average of 62% more contrast than healthy subjects in all spatial frequencies tested and an average of 39.52% more chromatic contrast to detect stimuli. It may be concluded that changes in visual expression may be considered a biological marker to distinguish the onset of Hansen’s disease.

## Data Availability

The authors understand and declare the availability of empirical data.

## Abbreviations

VEP: Visual potentials evoked
CS: Contrast sensitivity
cpd: Cycle by visual angle degree
D15d: Lanthony D-15 desaturated test
CCI: Color confusion index
CCT: Cambridge Color Test
CSF: Contrast sensitivity function

## References

[CR1] Andrade, M. J. O., Neto, A. C., Oliveira, A. R., Santana, J. B., & Santos, N. A. (2018a). Daily variation of visual sensitivity to luminance contrast: effects of time of measurement and circadian typology. *Chronobiology International*, *35*(7), 996–1007. 10.1080/07420528.2018.1450753. 10.1080/07420528.2018.1450753 29565681

[CR2] Andrade, M. J. O., Santos, L. G. B., Cristino, E. D., Oliveira, A. R., & Santos, N. A. (2018b). Daily variation of visual perception of colors: preliminary studies. *Psychology & Neuroscience*, *11*(3), 238–251. 10.1037/pne0000132.

[CR3] Bechler RG (2011). Reconhecendo Armauer Hansen: o cientista da lepra e o personagem histórico. Fronteiras.

[CR4] Brasil (2018). Situação epidemiológica e estratégias de prevenção, controle e eliminação das doenças tropicais negligenciadas no Brasil, 1995 a 2016. Boletim Epidemiológico.

[CR5] Chukka RK, Sheeladevi S, Pujari S (2011). Visual impairment in patients with leprosy in Adilabad district in the state of Andhra Pradesh, India. Indian Journal Leprosy.

[CR6] Costa TH, Barboni MTS, Moura ALA, Bonci DMO, Gualtieri M, Silveira LCL, Ventura DF (2012). Long-term occupational exposure to organic solvents affects color vision, contrast sensitivity and visual fields. PLoS ONE.

[CR7] Courtright, P., Daniel, E., Sundarrao et al (2002). Eye disease in multibacillary leprosy patients at the time of their leprosy diagnosis: findings from the Longitudinal Study of Ocular Leprosy (LOSOL) in India, the Philippines, and Ethiopia. *Leprosy Review*, *73*, 225-238. 12449887

[CR8] Daniel E, Sundary T, Appavoo R, Chacko S, Ragupathy A, Raju R (2005). Impaired contrast sensitivity among leprosy patients with normal visual acuity. Leprosy Review.

[CR9] Dethlefs R (1981). Prevalence of ocular manifestations of leprosy in Port Moresby, Papua New Guinea. Brazilian Journal Ophthalmology.

[CR10] Eballe AO, Owono D, Book AU, Bella AL, Mvoqo CE, Mba N (2009). Prevalence and etiologies of visual handicaps in leprosy patients in the south of Cameroon. Clinic Ophthalmology.

[CR11] Geller AM (2001). A table of color distance scores for quantitative scoring of the Lanthony desaturate color vision test. Neurotoxicology and Teratology.

[CR12] Geller AM, Hudnell HK (1997). Critical issues in the use and analysis of the Lanthony Desaturate Color Vision test. Neurotoxicology and Teratology.

[CR13] Lanthony P, Dubois-Poulsen A (1973). Le Farnsworth—15 désaturé [Desaturated Farnsworth 15]. Bulletin des societes d’ophtalmologie de France.

[CR14] Lima MG, Gomes BD, Ventura DF, Silveira LCL (2011). Métodos utilizados na avaliação psicofísica de cores humana [Methods used in the psychophysical evaluation of human color vision]. Psicologia USP.

[CR15] MacLeod DI (2003). New dimensions in color perception. Trends in Cognitive Sciences.

[CR16] Malik ANJ, Morris RW, Ffytche TJ (2011). The prevalence of ocular complications in leprosy patients seen in the United Kingdom over a period of 21 years. Eye.

[CR17] Mendonça VM, Costa RD, Melo GEBA, Antunes C, Teixeira AL (2008). Immunology of leprosy. Anais Brasileiros de Dermatologia.

[CR18] Mollon, J. D. & Regan, J. P. (2000). *Cambridge Colour Test*. Handbook

[CR19] Neto JLA, Cunha AP, Barreto RBP, Arantes TEF, Santos LPF (2006). Visual evoked potential in leprosy patients. Arquivo Brasileiro Oftalmologia.

[CR20] Rambukkana A (2000). How does Mycobacterium leprae target the peripheral nervous system?. Trends in Microbiology.

[CR21] Ribeiro MDA, Silva JCA, Oliveira SB (2018). Estudo epidemiológico da hanseníase no Brasil: reflexão sobre as metas de eliminação. *Revista Panamericana Salud Publica.* 42-42. doi:10.26633/RPSP.2018.42 10.26633/RPSP.2018.42 PMC6386138 31093070

[CR22] Santos NA, Simas MLB, Nogueira RMTBL (2003). Processamento Visual da Forma em Idosos: Curvas de Limiar de Contraste para Freqüências Angulares e Senoidais. Psicologia: Reflexão e Crítica.

[CR23] Shrestha S, Shrestha C, Shrestha SM, Manoranjan A, Dhungana AP (2018). Ocular morbidity among leprosy patients at a leprosy centre. MJSBH.

[CR24] Simpson CA, Fonsêca LCT, Santos VRC (2010). Perfil do doente de hanseníase no estado da Paraíba. Hansenologia Internationalis.

[CR25] Souza FS, Almeida LNF, Costa JP, Rocha PV, Sobrinho EFA (2005). Frequency of ocular changes in patients with Hansen's disease living in a colony hospital. Arquivos Brasileiros de Oftalmologia.

[CR26] Thompson KJ, Allardice GM, Babu GR, Roberts H, Kerketta W, Kerketta A (2006). Patterns of ocular morbidity and blindness in leprosy-a three centre study in Eastern India. Leprosy Review.

[CR27] Ulvi H, Yigiter R, Yoldas T, Erdem I, Müngen B (2003). Study of visual evoked potentials in the assessment of the central optic pathways in leprosy patients. Neurol Science.

[CR28] Ventura R, Malta JSM, Lyra AFC, Danda DMR, Urbano LCV (2009). Associação de alterações cromáticas e uso de anticoncepcionais orais. Arq Bras Oftalmologia.

[CR29] Vieira CSCA, Soares MT, Ribeiro CTSX, Silva LFG (2008). Avaliação e controle de contatos faltosos de doentes com hanseníase. Revista Brasileira Enfermagem.

[CR30] Whitcher JP, Srinivasan M, Upadhyay MP (2001). Corneal blindness: a global perspective. Bulletin of the World Health Organization.

